# Human mitochondrial ferritin improves respiratory function in yeast mutants deficient in iron–sulfur cluster biogenesis, but is not a functional homologue of yeast frataxin

**DOI:** 10.1002/mbo3.18

**Published:** 2012-06

**Authors:** Robert Sutak, Alexandra Seguin, Ricardo Garcia-Serres, Jean-Louis Oddou, Andrew Dancis, Jan Tachezy, Jean-Marc Latour, Jean-Michel Camadro, Emmanuel Lesuisse

**Affiliations:** 1 Department of Parasitology, Faculty of Science, Charles University in PragueVinicna 7, 128 44 Prague, Czech Republic; 2 Laboratoire Mitochondries, Métaux et Stress oxydant, Institut Jacques Monod, CNRS-Université Paris DiderotFrance; 3 CEA, iRTSV, LCBM, 38054 Grenoble Cedex 9, France; CNRS, UMR5249, Grenoble, France, Université Joseph Fourier38054 Grenoble Cedex 9, France; 4 University of Pennsylvania,, Department of Medicine, Division of Hematology/OncologyBRBII Room 731, 431 Curie Blvd, Philadelphia, PA 19104

**Keywords:** Ferritiny, ggc1, iron, mitochondria, mitochondrial, Mössbauer, yeast frataxin, yfh1

## Abstract

We overexpressed human mitochondrial ferritin in frataxin-deficient yeast cells (Δ*yfh1*), but also in another mutant affected in [Fe-S] assembly (Δ*ggc1*). Ferritin was correctly processed and expressed in the mitochondria of these cells, but the fraction of total mitochondrial iron bound to ferritin was very low, and most of the iron remained in the form of insoluble particles of ferric phosphate in these mitochondria, as evidenced by gel filtration analysis of the mitochondrial matrix (fast protein liquid chromatography [FPLC]) and by Mössbauer spectroscopy. Mutant cells in which ferritin was overexpressed still accumulated iron in the mitochondria and remained deficient in [Fe-S] assembly, suggesting that human mitochondrial ferritin is not a functional homologue of yeast frataxin. However, the respiratory function was improved in these mutants, which correlates with an improvement of cytochrome and heme synthesis. Overexpression of mitochondrial ferritin in [Fe-S] mutants resulted in the appearance of a small pool of high-spin ferrous iron in the mitochondria, which was probably responsible for the improvement of heme synthesis and of the respiratory function in these mutants.

## Introduction

Friedreich's ataxia is the most common hereditary recessive ataxia. The gene responsible for this neurodegenerative disease was cloned in 1996 (Campuzano et al. 1996). The corresponding protein (frataxin) was found to be a small, highly conserved mitochondrial protein, the precise role of which is still controversial (for recent reviews, see Santos et al. 2010; Koeppen 2011). Frataxin deficiency has frequently been associated with abnormalities of iron metabolism (reviewed in Marmolino 2011), and it is now generally accepted that frataxin (Yfh1p in yeast) is one of the components of the mitochondrial [Fe-S] cluster machinery, acting either as an iron donor or more generally as a chaperone interacting in an iron-dependent manner with the desulfurase/scaffold proteins involved in [Fe-S] assembly (reviewed in Stemmler et al. 2010), maybe via allosteric activation of the [Fe-S] assembly complex (Bridwell-Rabb et al. 2011). However, a general consensus on the role of frataxin has not yet been reached (Bayot et al. 2011).

Among the different hypotheses that were proposed regarding the role of frataxin, several studies insisted on the ferroxidase activity of frataxin (Park et al. 2002, 2003), and on its capacity to oligomerize and to store mitochondrial iron in a similar way than ferritin, thereby detoxifying mitochondrial iron (O'Neill et al. 2005; Gakh et al. 2006). These observations lead several authors to consider frataxin as a mitochondrial iron storage protein (Gakh et al. 2002). This hypothesis was strengthened by the observation that human mitochondrial ferritin strongly improves viability of frataxin-deficient yeast cells (Campanella et al. 2004, 2009).

In a recent study, we provided evidence that yeast frataxin is not an iron storage protein in vivo (Seguin et al. 2010). As others, we observed frataxin oligomerization under different physiological conditions, but we never found iron being associated in vivo to frataxin oligomers (Seguin et al. 2009, 2010). Moreover, we showed that several different mutants affected in [Fe-S] assembly overaccumulate iron in the mitochondria in the same precipitated form, regardless of the presence/abundance of frataxin (Seguin et al. 2010). Our conclusion that frataxin is not an iron storage protein in vivo seemed to be inconsistent with the findings according to which mitochondrial human ferritin rescues respiratory function and resistance to oxidative stress in yeast frataxin-deficient cells (Campanella et al. 2004, 2009). In our previous work (Seguin et al. 2010), we argued that ferritin expression might improve cell viability by making mitochondrial iron more available for biological functions in mutants which overaccumulate iron in the mitochondria in an insoluble form, that is, in all yeast mutants deficient in [Fe-S] biogenesis (Miao et al. 2008, 2009; Seguin et al. 2010). We checked this hypothesis in the present work by studying the effect of human mitochondrial ferritin overexpression in two different yeast mutants affected in [Fe-S] biogenesis for different causes (lack of frataxin in the Δ*yfh1* mutant, lack of the GTP/GDP carrier in the Δ*ggc1* mutant). We conclude that yeast frataxin is not a functional homologue of human mitochondrial ferritin, even though the presence of ferritin in the mitochondria of [Fe-S] yeast mutants improves their respiratory function.

## Material and Methods

### Yeast strains and growth conditions

The strains used in this study were YPH499 (wild-type (WT); MATa *ura3*-*52 lys2*-*801 ade2*-*101 trp1*-Δ*63 his3*-*Δ200 leu2*-Δ*1 cyh2*), YPH499Δ*yfh1* (*Δyfh1*; Δ*yfh1*::*TRP1*), YPH501Δ*yfh1* (MAT*α ura3-52 lys2-801 ade2-101 trp1-63 his3-200 leu2-1 cyh2 yfh1::HIS3* [p*GAL1-YFH1-URA3*], and YPH499Δ*ggc1* (MAT*a ura3-52 lys2-801 ade2-101 trp1-63 leu2-1 cyh2 ggc1*::*HIS3*). For overexpression of human mitochondrial ferritin, these strains were transformed by the plasmid pG3-MtF/TRP1, kindly provided by Dr S. Levi (high-copy expression plasmid bearing the cDNA for the full-length precursor of human mitochondrial ferritin under the control of the strong promoter of glyceraldehyde-3-phosphate dehydrogenase [Campanella et al. 2004]). For overexpression of frataxin (Yfh1p) in Δ*ggc1* cells, we used the high-copy plasmid pRS423 bearing *YFH1* under control of its native promoter, as previously described (Seguin et al. 2009). To avoid the accumulation of suppressor mutations in frataxin-deficient cells, the *yfh1* deletion was covered by a shuffle plasmid bearing a WT copy of *YFH1* and *URA3*. The covering plasmid was ejected before each experiment by plating the cells on minimum medium containing 5-fluoroorotic acid in anaerobic conditions. Unless otherwise stated, cells were grown aerobically in defined medium (6.7 g/L yeast nitrogen base without amino acids, iron or copper, and containing 0.1% glucose, 2% raffinose, 0.8 g/L amino acids). The defined media were supplemented with 5 μM CuSO_4_ and with various amounts of iron, in the form of ferric citrate.

### Cell fractionation

Mitochondria were isolated after treatment of cells with zymolyase, followed by lysis of the protoplasts in 0.6 M sorbitol (buffered with TRIS 50 mM, pH 7.8), in the presence of protease inhibitors (Protease inhibitor cocktail, P8215, Sigma) as described previously (Raguzzi et al. 1988). Submitochondrial fractionation was carried out as previously described (Martin et al. 1998), by hypotonic shock followed by sonication (1 min, three times) in 10 mM HEPES buffer (without EDTA; pH 7.2).

### Mössbauer spectroscopy

^57^Fe Mössbauer experiments were operated using a 50 mCi source of 57Co(Rh) (AREVA). The spectra were recorded either on a zero-field Mössbauer spectrometer equipped with a custom-made cryostat or on a strong-field Mössbauer spectrometer equipped with an Oxford Instruments Spectromag 4000 cryostat containing an 8T split-pair superconducting magnet. Both spectrometers were operated in a constant acceleration mode in a transmission geometry. The 14.4 keV γ-rays were detected by means of a proportional counter and the spectra were recorded on a 512 multichannel analyzer working in the multiscaling mode. The temperature of the sample was measured with a Pt resistor. The system was calibrated with a metallic iron foil absorber at room temperature, and all velocity scales and isomer shifts are referred to the iron standard. Analysis of the data was performed with the program WMOSS (WEB Research, Edina, MN).

### Iron accumulation and enzyme assays

Iron accumulation was measured after growing cells overnight in minimum medium with 1-10 μM ^55^Fe(III)-citrate. The specific activity of iron was 29,600 MBq/mg. Aconitase activity was measured in isolated mitochondria, according to a published method (Kennedy et al. 1983).

### Fast protein liquid chromatography (FPLC)

The distribution of iron-containing proteins from the mitochondrial matrix was analyzed by size-exclusion chromatography. Mitochondria (from cells grown with 1 μM ^55^Fe(III)-citrate) were lysed in hypotonic buffer (HEPES 10 mM, pH 7) and sonicated for 30 sec before being centrifuged at 10,000 *g* for 15 min. Supernatants (0.5 mL, 20 mg protein/mL) were loaded onto a Superdex 200 10/300 GL column (GE Healthcare) and proteins were eluted at a flow rate of 0.5 mL/min with 50 mM HEPES and 140 mM NaCl, pH 8.0, using the FPLC system ÄKTA purifier UPC 10 (GE Healthcare). Fractions (0.4 mL) were collected and the radioactivity of each fraction was counted in a microplate scintillation counter (MicoBeta TriLux). The column was calibrated with a gel filtration standard (Bio-Rad).

### Determination of heme synthesis in isolated mitochondria

Ferrochelatase, zinc-chelatase, and protoporphyrinogen oxidase activities were measured fluorimetrically, as previously described (Camadro and Labbe 1988; Camadro et al. 1994). Protoporphyrinogen was prepared from protoporphyrin by reduction with a sodium amalgam (Camadro et al. 1986). Endogenous mitochondrial iron/zinc availability to ferrochelatase was measured in isolated mitochondria (100 μg/mL), using protoporphyrinogen (2 μM) as a substrate, as previously described (Lesuisse et al. 2003). We followed the rates of PPIX (protoporphyrin IX; λexc = 410 nm; λem = 632 nm) and Zn-PPIX (λexc = 420 nm; λem = 587 nm) formation simultaneously. The rate of heme synthesis was calculated as total PPIX (measured fluorimetrically in the presence of 1 mM EDTA) minus (Zn-PPIX + PPIX) (measured fluorimetrically without EDTA).

### Other

The respiratory activity of isolated mitochondria was evaluated by an oxypolarographic method. The rate of oxygen consumption was measured with a 1-mL thermostatically controlled oxypolarographic cell equipped with a Clark-type electrode. The respiratory medium was 0.6 M sorbitol buffered with 0.1 M potassium phosphate (pH 7.2), saturated with air at 30°C (236 μM dissolved O_2_). The electron donor for respiration was NADH (1 mM).

Low-temperature spectra (–191°C) of whole cells were recorded as previously described (Labbe and Chaix 1969, 1971).

## Results

### Expression of human mitochondrial ferritin in yeast

**Figure 1:**
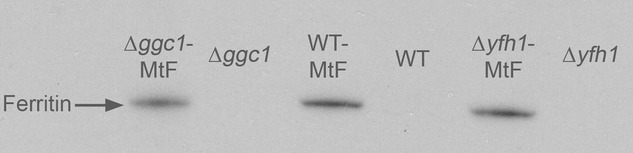
Expression of human mitochondrial ferritin in yeast mitochondria. Wild-type (WT) Δ*ggc1* and Δ*yfh1* cells transformed or not by pG3-MtF/TRP1 (MtF) were grown overnight in minimum medium containing 1 μM ^55^Fe(III) citrate as iron source. Mitochondria were isolated, lysed in hypotonic buffer containing 1% Triton X-100, and heated at 70°C for 10 min. These extracts were centrifuged at 10,000 *g* for 5 min, and the supernatants were subjected to native PAGE (8%) before autoradiography of the gel. One single band appeared in the upper part of the gel only in mitochondria from cells transformed by pG3-MtF/TRP1 (MtF).

**Figure 2:**
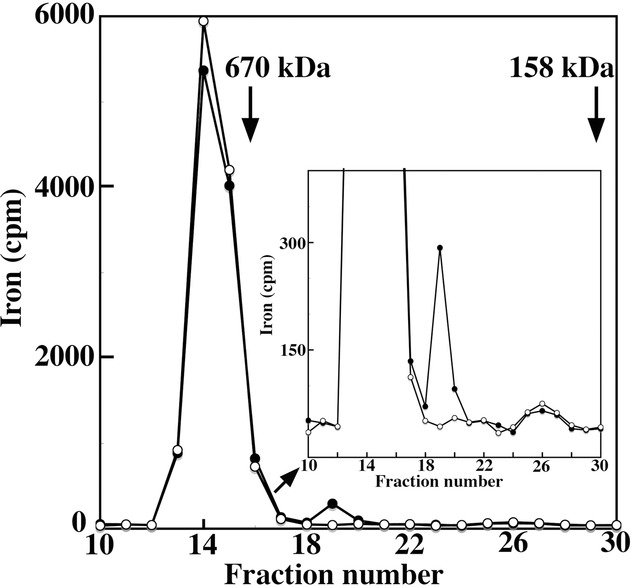
Analysis by gel filtration (fast protein liquid chromatography [FPLC]) of the mitochondrial matrix from Δ*yfh1* cells transformed (closed circles) or not (open circles) by pG3-MtF/TRP1. Cells were grown overnight with 1 μM ^55^Fe(III) citrate as iron source. Mitochondria were purified, subjected to hypotonic shock, and sonicated before being centrifuged. Supernatants (1-mg protein of mitochondrial matrix) were loaded onto a Superdex 200 column, and elution fractions were analyzed for ^55^Fe content by scintillation counting. Only high molecular weight fractions (in the range >670 kDa–158 kDa) were collected (standard molecular weight markers are indicated by arrows). Results are given in cpm per elution fraction (0.4 mL). Insert: enlargement of the region where ferritin eluted. The ferritin peak eluted at about 550 kDa, as evaluated by linear regression.

**Figure 3:**
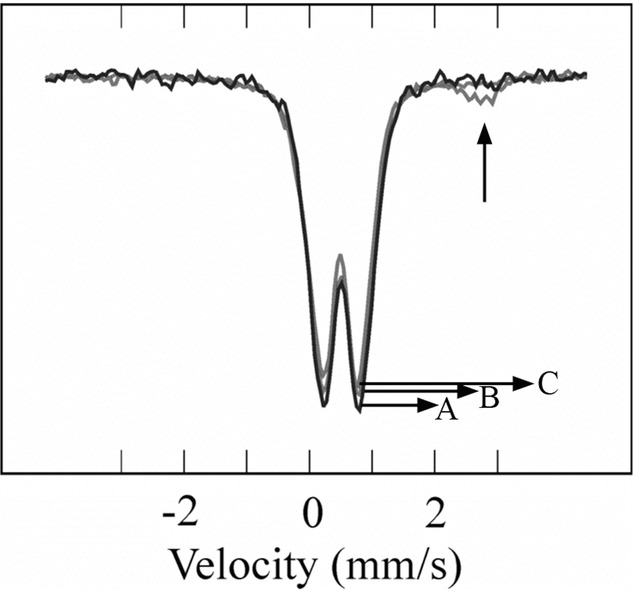
Mössbauer spectra of mitochondria purified from Δ*ggc1* cells (A), Δ*ggc1* cells overexpressing frataxin (B), and Δ*ggc1* cells overexpressing human mitochondrial ferritin (C). Spectra recorded at 78 K in zero-applied magnetic field. The peak of high spin Fe(II) (appearing only in spectrum C) is indicated by an arrow.

We overexpressed the full-length precursor of human mitochondrial ferritin in WT cells and in two yeast mutants affected in [Fe-S] cluster biogenesis (Δ*yfh1* and Δ*ggc1* strains) by transforming these strains with the plasmid pG3-MtF/TRP1, which was constructed and used by Campanella and coworkers (2004) in a previous study. Yeast Δ*yfh1* cells lack frataxin and Δ*ggc1* cells lack the mitochondrial GTP/GDP carrier (Vozza et al. 2004) that is required for mitochondrial [Fe-S] biogenesis (Amutha et al. 2008). We previously reported that both mutants accumulate iron in the mitochondria in the same form of amorphous nanoparticles of ferric phosphate (Seguin et al. 2010). Campanella and coworkers (2004) showed that the human mitochondrial ferritin encoded by the plasmid pG3-MtF/TRP1 was efficiently imported by yeast mitochondria and processed to functional ferritin that actively sequestered iron in the organelle. We checked that this result was reproducible under our experimental conditions by purifying mitochondria of cells transformed or not by pG3-MtF/TRP1 and grown in the presence of ^55^Fe(III)-citrate. The mitochondria were lysed and subjected to heat denaturation (70°C) before analysis of iron distribution in the remaining proteins by native gel electrophoresis followed by autoradiography of the gel (Fig. 1). Our results clearly show that an iron-binding protein resistant to heat denaturation (ferritin) was present in mitochondria of cells transformed by pG3-MtF/TRP1 (Fig. 1), in agreement with previous published data (Campanella et al. 2004). An important point raised by this result is to assess quantitatively the relative amount of mitochondrial iron bound to ferritin (vs. total mitochondrial iron) when this protein was overexpressed in mutants affected in [Fe-S] biogenesis. In their study, Campanella et al. (2004) determined that expression of mitochondrial ferritin in Δ*yfh1* cells prevented mitochondrial iron accumulation by these cells. We were not able to reproduce this last result: in our hands, expression of human mitochondrial ferritin in either Δ*yfh1* or Δ*ggc1* cells did not significantly decreased total mitochondrial iron accumulation by the cells: iron uptake rates by the cells in exponentially growth phase, as measured with 1 μM ^55^Fe(II)-ascorbate, were as follows (results given in picomoles per hour per million cells; means ± SE from four experiments): WT: 2.6 ± 0.1; WT-MtF: 2.9 ± 0.3; Δ*yfh1*: 25 ± 3; Δ*yfh1*-MtF: 23 ± 2; Δ*ggc1*: 18 ± 2; Δ*ggc1*-MtF: 20 ± 3. Total mitochondrial iron in stationary phase cells grown in the presence of 10 μM ^55^Fe-citrate was in the range of 5–8 mM in Δ*yfh1* and Δ*ggc1* cells overexpressing or not ferritin, and about 10-fold lower in WT cells (regardless of ferritin expression). Moreover, the proportion of mitochondrial iron bound to ferritin (relative to total mitochondrial iron) was very small in both strains, as determined by FPLC analysis and by Mössbauer spectroscopy. FPLC analysis (in the range of high molecular weight proteins) of the mitochondrial matrix of Δ*yfh1* cells transformed or not by pG3-MtF/TRP1 and grown with 10 μM ^55^Fe(III)-citrate showed a main peak of iron associated with very high molecular weight markers (>670 kDa) (Fig. 2). As previously shown (Seguin et al. 2010), this peak probably corresponds to small particles/aggregates of iron that were not completely removed by centrifugation and to iron associated to high molecular weight protein complexes. This peak was similar in the mitochondrial matrix of cells overexpressing ferritin or not (Fig. 2). A second, very small peak appeared only in the matrix of ferritin-overexpressing cells, and most probably corresponded to mitochondrial ferritin (molecular weight around 550 kDa; Fig. 2). This peak represented less than 0.5% of total iron present in the mitochondrial matrix of Δ*yfh1* cells overexpressing ferritin, which shows that ferritin-bound iron represented only a very small pool of iron in the mitochondria. We also compared the pools or iron present in the mitochondria of Δ*ggc1* cells and of Δ*ggc1* cells overexpressing either yeast frataxin or human mitochondrial ferritin by Mössbauer spectroscopy (Fig. 3). In a previous work (Seguin et al. 2010), we examined the form of iron accumulation in mitochondria of Δ*ggc1* cells. We have now compared iron accumulation by this strain to that accumulated in mitochondria of cells with the same deletion, but overexpressing either frataxin or ferritin, in order to search for any difference in the nature of the accumulated iron. Figure 3 illustrates the Mössbauer spectra of the mitochondria at 77 K. As already observed for the Δ*ggc1* cells, the Mössbauer spectrum is dominated by an intense quadrupole doublet with δ = 0.52(1) mm/sec, ΔE_Q_ = 0.63(2) mm/sec, and Γ = 0.52/0.50(2) mm/sec, typical of a high-spin ferric iron bound to oxygen/nitrogen in an octahedral arrangement that is associated to nanoparticles of ferric phosphate (Lesuisse et al. 2003; Seguin et al. 2010). Mitochondria of Δ*ggc1* exhibited the same spectrum, whether or not frataxin (Yfh1p) was overexpressed (Fig. 3). The spectrum of mitochondria from Δ*ggc1* cells overexpressing ferritin was mainly identical to the spectra of samples without ferritin overexpression, with however an additional small peak at δ = 2.9(1) mm/sec in addition to the major doublet, which corresponds to the high-energy line of a quadrupole doublet with δ ≍ 1.23 mm/sec and ΔE_Q_ ≍ 2.98, accounting for about 8% of total iron (Fig. 3). Such parameters are characteristic of a high-spin Fe(II) ion in an octahedral oxygen/nitrogen environment. Owing to the low intensity and to the broadness of this peak, the presence of several Fe (II) species cannot be excluded. According to its isomer shift, it can be associated to either ferrous ion bound to the ferroxidase site of ferritin (Watt et al. 1985; Yang et al. 1987) or “free” Fe(II) ions constituting the so-called “labile iron pool” (Garber-Morales et al. 2010). However, FPLC analysis of ^55^Fe-labeled mitochondria showed that iron bound to ferritin did not exceed 0.5% of total mitochondrial iron (Fig. 2). Therefore, our results suggest that most of the Fe(II) species detected by Mössbauer belongs to a labile pool of ferrous iron, similar to that evidenced by others (Garber-Morales et al. 2010).

As we did in our previous study (Seguin et al. 2010), we measured the spectra of mutants overexpressing ferritin at temperatures down to 2 K, not seeing evidence of a magnetic transition (see Fig. S1). In mammalian ferritins, the presence of a magnetically ordered phase is the identifying feature of highly crystalline ferric oxyde/hydroxyde cores. The absence of such a trait is therefore indicative that the iron accumulated in the mitochondria overexpressing ferritin was not mainly stored in the ferritin core, but massively remained precipitated as amorphous nanoparticles of iron phosphate. However, it must be kept in mind that the phosphate content is a key factor in the magnetic behavior of ferritin iron cores, those with higher phosphate contents having lower paramagnetic transition temperatures (St Pierre et al. 1996).

### Human mitochondrial ferritin improves respiratory function in [Fe-S] mutants

**Figure 4:**
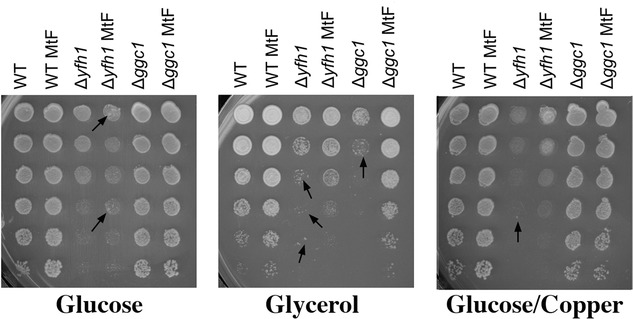
Effect of human mitochondrial ferritin overexpression (MtF) on the growth of wild-type (WT) Δ*yfh1* and Δ*ggc1* cells on plates (serial dilutions) with glucose or glycerol as the carbon source, or with 0.5 mM copper (CuSO_4_, glucose as the carbon source). Note the appearance of numerous suppressor colonies (arrows) especially when Δ*yfh1* and Δ*ggc1* cells were plated on a nonfermentable carbon source (glycerol).

**Figure 5:**
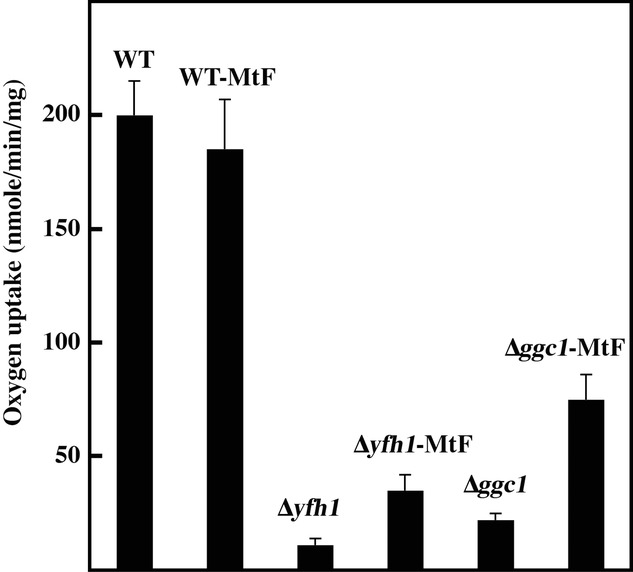
Oxygen consumption by mitochondria isolated from wild-type (WT) Δ*ggc1* and Δ*yfh1* cells transformed or not by pG3-MtF/TRP1 (MtF). Isolated mitochondria were incubated at 30°C in the chamber of a Clark-type electrode under magnetic stirring in the presence of 1 mM NADH, and the initial slope of oxygen uptake was recorded. Mean ± SE from three experiments.

In their work, Campanella et al. (2004) reported that Δ*yfh1* cells overexpressing ferritin recovered a WT level of respiration and were more resistant than Δ*yfh1* cells to oxidative stress. We also found that expression of human mitochondrial ferritin improved the ability of Δ*yfh1* cells to grow on a nonfermentable carbon source, and increased the resistance of cells to copper-mediated oxidative stress (Fig. 4). However, this phenotype was not restricted to Δ*yfh1* cells, since overexpression of mitochondrial ferritin also improved the capacity of Δ*ggc1* cells to grow on a nonfermentable carbon source, even better than when ferritin was expressed in Δ*yfh1* cells (Fig. 4). The [Fe-S] mutants overexpressing ferritin also had a higher rate of oxygen consumption than untransformed cells, consistent with an improvement of the respiratory function in these cells (Fig. 5). The improvement of the respiratory function in Δ*yfh1* and Δ*ggc1* cells by ferritin expression was not due to a restoration of the [Fe-S] biogenesis in these cells, since overexpression of mitochondrial ferritin did not change significantly aconitase activity in either Δ*yfh1* or Δ*ggc1* cells: aconitase activity in these cells was about 10% of that found in WT cells, with or without overexpression of mitochondrial ferritin, as shown by the following data (aconitase activity

expressed in nanomoles per min per milligram mitochondrial protein; mean ± SE from three experiments): WT: 25 ± 2.4; WT-MtF: 31.4 ± 8; Δ*yfh1*: 2.7 ± 0.2; Δ*yfh1*-MtF: 2.2 ± 0.3; Δ*ggc1*: 2.8 ± 0.9; Δ*ggc1*-MtF: 3.5 ± 0.9.

**Figure 6:**
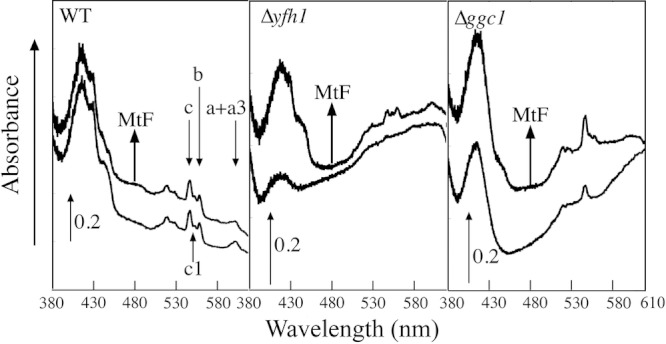
Cytochrome content of the cells. Wild-type (WT) Δ*ggc1* and Δ*yfh1* cells transformed or not by pG3-MtF/TRP1 (MtF) were grown overnight in minimum medium with raffinose as the carbon source, harvested and washed with water before recording low-temperature spectra of whole cells.

**Figure 7:**
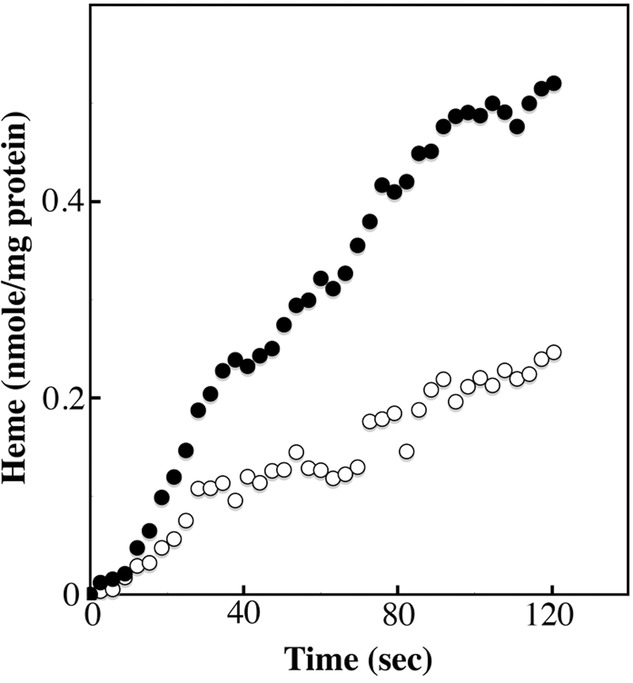
Heme synthesis from endogenous iron in isolated mitochondria from Δ*yfh1* cells transformed (closed circles) or not (open circles) by pG3-MtF/TRP1. Mitochondria were isolated from the cells and incubated at 100 μg/mL in isotonic buffer (0.5 M sorbitol, 0.1 M TRIS, pH 7.4). The reaction was initiated by adding 2 μM protoporphyrinogen to the suspension of mitochondria (see Section Methods). Results are from one representative experiment.

Cells affected in [Fe-S] biogenesis are also deficient for heme synthesis, due to metabolic remodeling (Hausmann et al. 2008), and because iron precipitates in the mitochondria of these cells and is thus unavailable to ferrochelatase (Lesuisse et al. 2003). Since the defect in heme and cytochrome synthesis should contribute to the phenotype of respiration deficiency in mutants affected in [Fe-S] biogenesis, we checked whether or not human ferritin expression could improve respiration in Δ*yfh1* and Δ*ggc1* cells through some restoration of heme/cytochrome synthesis. Our data show that, indeed, cytochrome synthesis in vivo was improved by ferritin overexpression in both Δ*yfh1* and Δ*ggc1* cells (Fig. 6). The level of cytochromes produced by these cells did not reach, however, WT levels (Fig. 6). This effect was probably due to iron being more available to ferrochelatase in the mitochondria, since in vitro heme synthesis by isolated mitochondria from endogenous iron and exogenous protoporphyrinogen was also improved by ferritin overexpression (Fig. 7). We conclude that overexpression of human mitochondrial ferritin rescues some respiratory functions in frataxin-deficient cells, as previously reported by others (Campanella et al. 2004), but that this effect is not specific since it is also observed in another mutant affected in [Fe-S] biogenesis, in which frataxin is present (Δ*ggc1* mutant). Moreover, our data suggest that mitochondrial ferritin does not act as a functional homologue of frataxin, but rather acts nonspecifically by making a small iron pool of the mitochondria available for heme synthesis in cells where mitochondrial iron precipitates in an amorphous form unavailable for biological functions.

## Discussion

It has been long recognized that yeast cells lacking frataxin accumulate iron in the mitochondria (Babcock et al. 1997; Foury and Cazzalini 1997). This finding gave rise to strong hope in the scientific community regarding the possibility to treat Friedreich ataxia patients by iron chelators (Richardson 2003). However, it is now clear that all yeast mutants affected one way or another in [Fe-S] biogenesis accumulate iron in the mitochondria, regardless of the presence of frataxin, and that this iron is biologically unavailable because it precipitates in the form of nanoparticles of ferric phosphate (Miao et al. 2008, 2009, 2011; Seguin et al. 2010). It is now a big challenge to understand why iron accumulated by the cells as a result of [Fe-S] biogenesis deficiency ends up in the mitochondria, whereas iron accumulated by the cells as a result of a constitutive activation of the iron uptake systems (*AFT1*up mutant) accumulates in the normal iron storage compartment, the vacuole (Miao et al. 2011). Also, several phenotypes that were previously presented as specifically related to the lack of frataxin are now recognized as being shared by other [Fe-S] mutants, in particular the phenotypes of mitochondrial iron accumulation and of heme deficiency (Hausmann et al. 2008). This is the reason why we proposed that, as far as possible, results obtained with frataxin-deficient yeast cells should not only be compared with those obtained in WT control cells, but also in some other mutant(s) affected in [Fe-S] biogenesis, in which frataxin is present (Seguin et al. 2010). This is what we did in the present study. As previously reported by others (Campanella et al. 2004, 2009), we observed that overexpression of human mitochondrial ferritin in frataxin-deficient cells (Δ*yfh1* mutant) resulted in some improvement of the respiratory function. However, the same result was obtained with the Δ*ggc1* mutant, which implies that the effect of ferritin was not specific and not restricted to frataxin-deficient cells. Moreover, we were not able to reproduce several important results described in the previous studies of Campanella and coworkers. In particular, in our hands ferritin did not rescue [Fe-S] biogenesis and did not change the phenotype of mitochondrial iron accumulation in either Δ*yfh1* or Δ*ggc1* mutants. Rather, we showed that human mitochondrial ferritin could bind a very small fraction of the iron accumulated in the mitochondria of these mutants, and that most of the iron accumulated in the mitochondria remained in a precipitated form not available to mitochondrial ferritin. A similar observation was made in ABCB7-deficient HeLa cells (Cavadini et al. 2007), which is consistent with the present results: ABCB7 is the human ortholog of Atm1p, and Δ*atm1* cells accumulate iron in the same form than Δ*yfh1* and Δ*ggc1* cells in the mitochondria (Miao et al. 2009; Seguin et al. 2010). In our view, these observations indicate that yeast frataxin is not a functional homologue of human mitochondrial ferritin. We cannot readily explain the discrepancies between our results (present work) and those published previously (Campanella et al. 2004, 2009). We reproduced our results several times in independent experiments, where we transformed repeatedly the Δ*yfh1* and Δ*ggc1* mutants by the same plasmid used by Camapanella and coworkers (2004) in their studies to overexpress human mitochondrial ferritin. In their work, Δ*yfh1* cells transformed by pG3-MtF/TRP1 (ferritin overexpression) recovered a WT phenotype, which is a very striking result. In our experience, each time Δ*yfh1* cells recovered a WT phenotype, whatever the experimental background, this was due to a suppressor mutation, which appears at a high rate especially under the selective pressure of growth in a nonfermentable carbon source (Lesuisse et al. 2003). This suppressor mutation was recently identified as a dominant point mutation in *ISU1* (Yoon et al. 2012). To avoid accumulation of suppressor mutations in Δ*yfh1* cells, we used a “shuffle strain” in which a covering plasmid bearing a WT copy of *YFH1* was ejected before each experiment (Seguin et al. 2011). Even with this precaution, we found several times that cultures of Δ*yfh1* cells, recovering a WT phenotype, did so due to suppressor mutation(s) and not due to the expression of human ferritin. Thus, one possible—and probably frequent—cause of discrepancy between the results obtained by different groups working on frataxin-deficient yeast cells is related to the occurrence of suppressor mutation(s).

Besides the discrepancies, some of our results are in agreement with those previously published (Campanella et al. 2004, 2009): the presence of human mitochondrial ferritin in the mitochondria of the [Fe-S] mutants improves the respiratory function of the cells, probably through improvement of heme/cytochrome synthesis. Respiration cannot occur without functional [Fe-S], the biogenesis of which was not improved by ferritin overexpression in our experiments. Presumably, the background level of [Fe-S] in Δ*yfh1* and Δ*ggc1* cells (evaluated to be about 10% of the WT level on the basis of aconitase activity) allowed the cells to better respire when the level of cytochromes was increased by ferritin overexpression. Improvement of heme synthesis in cells overexpressing human mitochondrial ferritin was most probably due to the presence in the mitochondria of these cells, of a pool of high-spin ferrous iron, which was not present in the absence of ferritin. In a remarkable study, Garber-Morales and coworkers (2010) recently showed that several distinct iron pools are present and interconnected in yeast mitochondria, the abundance of each pool depending on the cell metabolism (respiration or fermentation). According to the authors, the pool of nonheme high-spin ferrous iron serves as feedstock for [Fe-S] and heme synthesis (Garber-Morales et al. 2010). This pool of iron is absent in mitochondria of yeast mutants deficient in [Fe-S] assembly: most of the iron is in the form of nanoparticles of ferric phosphate in these mitochondria, and is therefore unavailable for biological functions (Lesuisse et al. 2003; Miao et al. 2009, 2011). The mechanism by which mitochondrial ferritin restores a small pool of labile ferrous iron is unclear. One possibility is that ferritin displaces and binds a small fraction of the mitochondrial iron precipitated as ferric phosphate. This ferric iron bound to ferritin could then be released (as Fe^2+^) from ferritin by reduction (more easily than from ferric phosphate), and could restore heme synthesis. Such a hypothesis remains to be confirmed. Why heme synthesis but not [Fe-S] assembly is restored by this iron pool is probably due to the fact that heme synthesis can be limited by the lack of available mitochondrial iron (Lesuisse et al. 2003), and restored by very low amounts of ferrous iron, given the very high affinity of ferrochelatase for Fe^2+^ (K_M_ = 0.16 μM) (Camadro and Labbe 1988). In contrast, restoration of [Fe-S] assembly would require ferritin being able to bypass specific defects of the [Fe-S] assembly machinery (lack of frataxin and lack of the GTP/GDP carrier in Δ*yfh1* and Δ*ggc1* strains, respectively), which is apparently not the case.

Thus, our work also clearly shows that human mitochondrial ferritin is not a functional homologue of the yeast frataxin. This result does not preclude, however, that frataxin might behave differently in other species. Although frataxin is a conserved protein found in most organisms, its function can be different according to the species: the *Escherichia coli* homologue CyaY (Iannuzzi et al. 2011) does not behave like the *Bacillus subtilis* Fra (Albrecht et al. 2011) or the yeast Yfh1p. Moreover, isoforms of frataxin in a same species might have different properties: in a recent study, Gakh and coworkers (2010) showed that one human frataxin isoform, with a shorter N-terminus, is involved in dynamic contacts with the [Fe-S] assembly machinery, whereas the isoform with a longer N-terminus would correlate with the ability of frataxin to oligomerize and store iron. This latter function of frataxin is probably not present in yeast.

## Supporting information

**Figure S1.** Mössbauer spectra of mitochondria from Δggc1-MtF (A and B) and Δssq1-MtF (C and D) yeast strains.
