# Supplementary material for: Human mitochondrial ferritin improves respiratory function in yeast mutants deficient in iron–sulfur cluster biogenesis, but is not a functional homologue of yeast frataxin
Source: Microbiologyopen. 2012 Jun;1(2):95–104. doi: 10.1002/mbo3.18 (PMC3426411; doi:10.1002/mbo3.18)
Supplement: Supplementary file 1 [file mbo30001-0095-SD1.pdf]

# Supplemental Figure

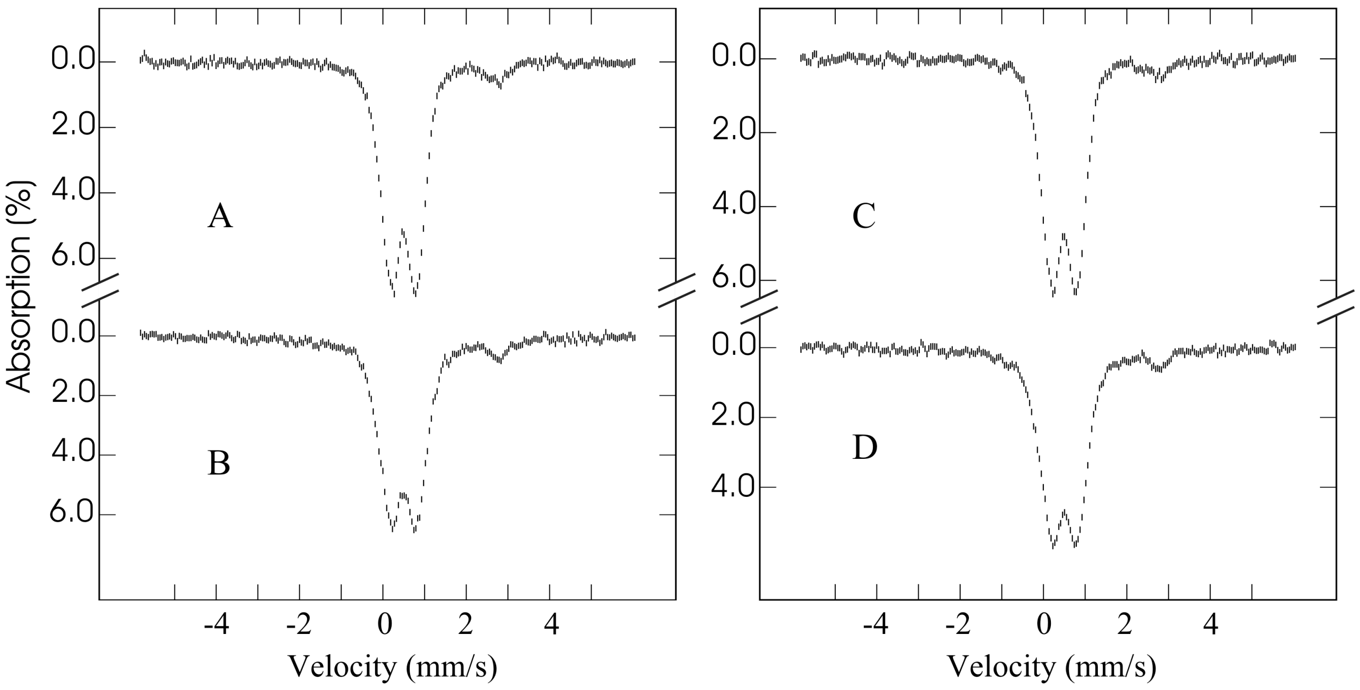


**Figure S1 :** Mössbauer spectra of mitochondria from Δggc1-MtF (A, B) and Δssq1-MtF (C, D) yeast strains. Spectra recorded at 77 K (A, C) or 4.2 K (B, D) in a 600 G magnetic field applied parallel to the gamma
