# Supplementary figures and images for: Human mitochondrial ferritin improves respiratory function in yeast mutants deficient in iron–sulfur cluster biogenesis, but is not a functional homologue of yeast frataxin
Source: Microbiologyopen. 2012 Jun;1(2):95–104. doi: 10.1002/mbo3.18 (PMC3426411; doi:10.1002/mbo3.18)

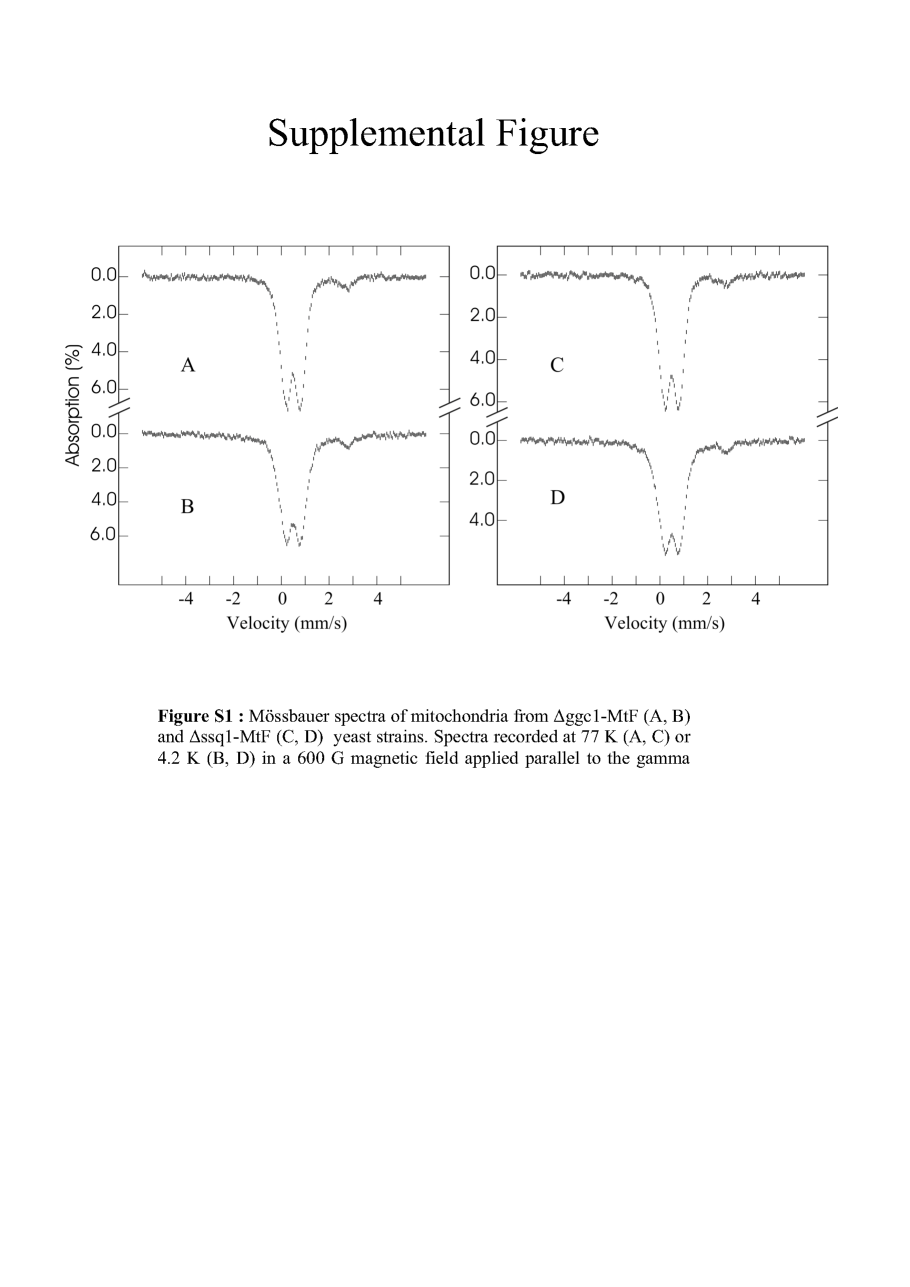

Supplement: Supplementary file 2 [file mbo30001-0095-SD2.png]
